# A meta-ethnographic synthesis on phenomenographic studies of patients’ experiences of chronic illness

**DOI:** 10.3402/qhw.v10.26279

**Published:** 2015-02-16

**Authors:** Marta Röing, Margareta Sanner

**Affiliations:** Department of Public Health and Caring Sciences, Health Services Research, University of Uppsala, Uppsala, Sweden

**Keywords:** Meta-ethnography, meta-synthesis, phenomenography, chronic illness, qualitative research

## Abstract

Phenomenography is a qualitative research approach developed within an educational framework, focusing on the qualitative experience of learning. It is also being used, to a lesser degree, in healthcare research. In the present study, we conducted a meta-ethnographic synthesis of phenomenographic studies on chronic illness, in order to give a broader perspective of how chronic illness can be experienced. Our aim was not to describe patients’ various individual experiences of illness, but instead to identify the different ways chronic illness can be experienced by patients. Our synthesis and phenomenographic interpretation of 12 selected articles found that patients’ experiences of chronic illness can be described in terms of a different lived body, a struggle with threat to identity and self-esteem, a diminished lifeworld, and a challenging reality. These experiences relate to each other in a process of recurring loops, where the different ways of experiencing continue to influence each other over time. According to these findings, the use of phenomenography as a research approach has the potential to add to the understanding of how chronic illness can be experienced. Patients may benefit from seeing that their illness can be experienced in many different ways and that it has many aspects, which then can lead to a better understanding and coping with their illness. We suggest that it may be worthwhile to expand the scope of phenomenography outside pedagogics. This presupposes a revision of the application to include a wider and more comprehensive description, for instance, of the different ways illness and healthcare phenomena can be experienced, and how these different ways are related to each other, with less focus on hierarchical relations.

Phenomenography is a qualitative research approach which was developed in the 1970s within an educational framework, focusing on the qualitative experience of learning.

Originally developed in Sweden, phenomenography studies the variations in ways that people look upon, experience, or understand phenomena in the world around them (Marton & Booth, 1997). It rests on a non-dualistic ontological perspective, that the real objective world and a subjective world of mental representations are not separate (Marton, 2000). Instead, the world is “simultaneously objective and subjective,” which means that an experience “is as much an aspect of the object as it is of the subject” (Marton, 2000, p. 105). In the same manner, a phenomenon, or object of experience, is seen as a complex of the different ways in which it can be experienced. These different ways are related to each other because they are experiences of the same object (Marton, 2000). Phenomenography is not to be mistaken for phenomenology, a well-established qualitative method used within healthcare research for many years, which investigates phenomena in order to reveal their structure or logic, and essence (Giorgi, 1999; Sjostrom & Dahlgren, 2002).

In a phenomenographic analysis of interview texts, ways of experiencing a phenomenon are identified and classified under categories of description which often form a hierarchy, and which eventually can be defined in terms of increasing complexity (Marton & Booth, 1997). In the last stage of the analysis, these categories are presented in an outcome space in such a way as to suggest the order and logical relation the different categories have to each other (Barnard, McCosker, & Gerber, 1999; Marton & Booth, 1997). This can be exemplified by one of the earliest phenomenographic studies carried out in Sweden, which searched for qualitative differences between individual students as to the outcome and process of their learning, based on their written reports after reading an academic text. The results revealed a hierarchy of categories in terms of depth of learning (from surface to deep learning), which reflected in turn the qualitatively different ways the students had comprehended the text (Richardson, 1999).

Notwithstanding this pedagogical focus, phenomenography has in recent years gained the interest of healthcare researchers as well, as, with its unique empirical approach, it has the potential of adding to knowledge within a healthcare framework (Barnard et al., 1999; Sjostrom & Dahlgren, 2002). In the work presented here, we were interested in the phenomenographic study of patients’ experiences of chronic illness.

The prevalence of chronic illnesses worldwide is increasing, as the result of better possibilities for treatment and survival (Wagner et al., 2001). Chronic illnesses are here defined as illnesses that are prolonged in duration, do not often resolve spontaneously, and are rarely cured completely. They may require repeated periods of treatment from healthcare providers and may also need to be managed by patients (Audulv, 2013; Wagner et al., 2001). Herein, based on this need to manage the illness, lies the potential of the phenomenographic approach in healthcare research, as the ways that patients experience their illnesses can be used as sources of knowledge in patient education and patient empowerment (Stattin, 2001). In learning to live with a chronic illness, patients need to understand not only their illness, but the way they experience it. Giving patients the opportunity to reflect upon their ways of experiencing can give them insight into their own needs and reactions, which then can lead to making decisions and taking control (Stattin, 2001).

In a preliminary exploratory search for previous research on chronic illnesses with a phenomenographic approach, we found a long series of studies, almost exclusively from Sweden. These studies, which dealt with different chronic illnesses, were each based on the experiences of few informants. Therefore, we decided to synthesize these studies using a meta-ethnographic method which allowed for an interpretation of the results within a phenomenographic framework. Thus, our aim was not to describe patients’ various individual experiences of illness, but instead to identify the different ways chronic illness can be experienced by patients.

## Method

The method chosen for our synthesis of research studies using a phenomenographic approach was meta-ethnography. This method, developed in 1988, is an inductive and interpretive form of knowledge synthesis (Noblit & Hare, 1988). It builds on identification of the original researchers’ interpretations, and development of new interpretations that go beyond those offered in the original studies (Campbell et al., 2011), and which then become the new data for the synthesis (Noblit & Hare, 1988). In this manner, the method was specifically suitable for our aim, to provide a new phenomenographic interpretation based on individual selected studies. The process of our synthesis included the following five phases:

1. Literature search for articles.
2. Selection of relevant articles after repeated reading and appraisal of the articles.
3. Extraction of data from each article and creating a list of findings as key phrases, ideas, and concepts for each individual study.
4. Determining how the findings of the selected studies are related and translating findings into one another.
5. Synthesizing the translations to produce a new phenomenographic interpretation.

The first phase (the literature search) was carried out by the first author. We then worked together in the following four phases of the synthesis, discussing complex and complicated issues until we came to an agreement.

### Phase 1. Literature search for articles

The initial search using the keyword “phenomenography” with no date restrictions began with the PubMed database because this database focuses mainly on medicine and biomedical sciences and is regularly updated with literature that has been presented online in an early version before print publication (Falagas, Pitsouni, Maliletzis, & Pappas, 2008). This search resulted in 35,431 articles. A preliminary check on the abstracts revealed that most of these articles dealt with learning, and phenomenography as a method was not even mentioned. Consequently, a search of PubMed, and even Cinahl, PsycINFO, and Scopus databases was continued using the keyword “phenomenographic approach.” At the time of the initial search, a total of 175 articles in the PubMed database had applied a phenomenographic approach in healthcare research, although with different aims (Table I).

**Table I:** Literature search of databases.

Database	Number of articles Keyword phenomenography	Number of articles Keyword phenomenographic approach
PubMed	35,431	175
Cinahl	36	115
PsycINFO	106	107
Scopus	482 (96 with subject area medicine)	380 (109 with subject area medicine)

Primary screening and selection criteria for articles applicable for a possible synthesis were that they focused on patients' experiences of chronic illness and used a phenomenographic approach to data collection and analysis. In total, 37 articles appeared to meet these criteria. With these in mind, a second search of the Cinahl, PsycINFO, and Scopus databases revealed the same articles as in PubMed, and no additional articles of interest.

### Phase 2. Selection of relevant articles after repeated reading and appraisal of the articles

After reading the abstracts from the original 37 articles we excluded all articles which did not deal with chronic illness, such as those dealing with patients’ experiences after recovery, or of old age, life changing situations (such as menopause and retirement), and so forth. Articles on mental disorders were also excluded, as we wished to focus on somatic diseases. Seventeen articles remained for further exploration and appraisal (Table II).

**Table II:** Articles selected for appraisal.

	Title	Country setting	Aim of study and number of participants
1.	Refusing to be ill: a longitudinal study of patients’ experiences of asthma/allergy (Scherman et al., 2002)	Sweden	To search for a deeper understanding of the ways in which patients with asthma/allergy experience their illness situation. 30 patients
2.	Living with chronic renal failure: patients’ experiences of their physical and functional capacity (Heiwe et al., 2003)	Sweden	To describe and analyse ways in which patients with chronic renal failure experienced their physical and functional capacity in their daily lives. 16 patients
3.	Physical activity in patients with venous leg ulcer—between engagement and avoidance. A patient perspective (Roaldsen et al., 2011)	Sweden	To identify and describe the qualitative variations in how physical activity is perceived and understood by individuals with current or previous leg ulcer. 22 individuals
4.	Patients’ perceptions of living with epilepsy a phenomenographic study (Raty & Wilde-Larsson, 2011)	Sweden	To describe how patients with epilepsy perceive living with epilepsy. 19 patients
5.	Hearing confirms existence and identity—experiences from persons with presbyacusis (Karlsson Espmark & Hansson Scherman, 2003)	Sweden	To describe how elderly persons with mild-to-moderate presbyacusis experience living with that type of hearing loss. 14 individuals
6.	Perceptions of health-related quality of life of men and women living with coeliac disease (Hallert et al., 2003)	Sweden	To describe and explore, using a qualitative approach, variations in the perception of HRQoL between men and women with long-standing coeliac disease. 10 patients
7.	Patients’ experiences of physical limitations in daily life activities when suffering from chronic heart failure: a phenomenographic analysis (Pihl et al., 2011)	Sweden	To describe how patients suffering from chronic heart failure (CHF) conceived their physical limitations in daily life activities. 15 patients
8.	Male patients with congestive heart failure and their conceptions of the life situation (Martensson et al., 1997)	Sweden	To describe, from a nurse's perspective, how male patients with CHF perceive their life situation. 10 patients
9.	Female patients with congestive heart failure: how they conceive their life situation (Martensson et al., 1998)	Sweden	To describe from a nurse's perspective, how female patients with CHF conceive their life situation. 12 female patients
10.	Stress in women's daily life before and after a myocardial infarction: a qualitative analysis (Sjostrom-Strand & Fridlund, 2007)	Sweden	To describe and explore women's perceptions of stress before and after an MI. 20 women before 14 women after
11.	Women's experience of a myocardial infarction: 5 years later (Sjostrom-Strand et al., 2011)	Sweden	To explore and describe how women conceived their health and daily life 5 years after an MI. 12 women
12.	Adapting to living with a mechanical aortic heart valve: a phenomenographic study (Oterhals et al., 2013)	Norway	To describe how patients adapt to living with a mechanical aortic heart valve. 20 patients
13.	How people with rheumatoid arthritis perceive leisure activities; a qualitative study (Wikstrom, Jacobsson, & Arvidsson, 2005)	Sweden	To explore how people with rheumatoid arthritis perceive leisure activities. 18 patients
14.	Views on Exercise maintenance: Variations among patients with Rheumatoid Arthritis (Swardh, Biguet, & Opava, 2008)	Sweden	To explore and describe ways of understanding exercise maintenance among individuals with RA who had already started to exercise. 14 women, 2 men
15.	The importance for daily occupations of perceiving good health: Perceptions among women with rheumatoid diseases (Ottenvall Hammar & Hakansson, 2013)	Sweden	To describe and characterize what women with rheumatoid arthritis (RA) and juvenile idiopathic arthritis (JIA) perceive as important in considering the performance of daily occupations to perceive good health. 9 women
16.	Patient's conception of coronary heart disease—a phenomenographic analysis (Karner, Goransson, & Bergdahl, 2003)	Sweden	To explore how patients with coronary heart disease view the nature of their disease. 23 patients
17.	Conceptions on treatment and lifestyle in patients with coronary heart disease—a phenomenographic analysis (Karner, Goransson, & Bergdahl, 2002)	Sweden	To investigate patients’ conceptions of their disease and treatment with focus on cognitive aspects of their drug treatment and lifestyle changes. 23 patients

After reading and rereading in full the 17 articles, we excluded two more articles (numbers 16 and 17 in Table II) as they were mostly physiologically or pharmacologically oriented. Three more articles (numbers 13 to 15 in Table II) were subsequently excluded during the synthesis, as we found the focus of these articles too narrow.

Our synthesis is thus based on the first 12 articles presented in Table II, representing 148 individuals. As can be seen, they are concerned with patients’ experiences of asthma/allergy, renal failure, epilepsy, coeliac disease, hearing impairment, and chronic heart diseases.

### Phase 3. Extraction of data from each article and creating a list of findings as key phrases, ideas, and concepts for each individual article

The process of translating the findings into each other began by rereading the final 12 articles and listing the original findings of each individual article (column 1, Table III). The first step involved a preliminary translation of the original titles to patients’ experiences, for each individual article (column 2, Table III), as we noted that the terms used in the original articles did not deal with experiences, but instead circumstances, or abstractions, etc. We then came to an agreement on further translation of these constructs into key ideas or phrases, again for each individual article (column 3, Table III). These were based not only on the results, but on the actual descriptions and citations in each individual article as well.

**Table III:** Phase 3: Extraction of data from each article and creating a list of findings as key phrases, ideas and concepts for each individual article.

	Article	1. Categories and subcategories (as presented by authors)	2. Translation to ways of experiencing (if necessary)	3. Further translation to key ideas/phrases
1.	Refusing to be ill: a longitudinal study of patients’ experiences of asthma/allergy (Scherman et al., 2002)	**What illness is** Knowing for oneself	Deciding myself if I am sick	Being vulnerable to environment Necessity of understanding own body Needing to take control Refusing to be completely steered by illness Wanting to preserve identity and self-esteem
		**How illness is caused** Body related Environment related Psychosomatic Magic Fatalism Compliance with medication Alternative medicine Health-care Provocation Avoidance	Understanding vulnerability of my body Understanding the unity of body and soul Believing in supernatural powers Taking medicine without questioning Taking control by searching and choosing for alternative cures Keeping self physically fit Building up resistance by provoking the body Avoiding triggering factors	
		**How illness can be cured** Normalisation Normification Pursuing life Main cat: Identity	Denying the illness Not wanting to be identified as a sick person Trying to carry on	
2.	Living with chronic renal failure: patients’ experiences of their physical and functional capacity (Heiwe et al., 2003)	**Fatigue** Mental domain Physical domain	Renal failure as mental fatigue Renal failure as physical fatigue	Limited capacity to live everyday life Overwhelming fatigue
		**Reduced functional capacity** Limited performanc Low endurance	Experiencing limited performance Experiencing low endurance	Constant lack of peace of mind
		**Temporal stress** Constant need of more time Constant lack of peace	Experiencing constant stress	
3.	Physical activity in patients with venous leg ulcer—between engagement and avoidance. A patient perspective (Roaldsen et al., 2011)	**Perception of venous leg ulcer as a chronic or acute condition** Self-management	Understanding the necessity of living with the disease and taking responsibility	Taking control of illness/ preferring not to be responsible
		**Engagement of avoidance behavior toward physical activity** Instructions and support Fear of injury Wish to stay normal	Requiring instructions and support Fearing pain and injury Wish to maintain identity as a normal person	Requiring support Fear Wanting to preserve identity
4.	Patients’ perceptions of living with epilepsy a phenomenographic study (Raty & Wilde-Larsson, 2011)	**Living with epilepsy is living a normal life—gaining and maintaining control** Accepting the person with epilepsy Taking responsibility Appreciating the good things	Accepting self with epilepsy Taking responsibility Appreciating the good things	Accepting/not accepting own chronic illness Being hindered from living a normal life
		**Living with epilepsy is living with focus on the condition—conflict and avoidance or resigning to fate** Struggling with feelings of stigma, prejudices and loss of control Giving up hope of recovery Accepting loss of control	Struggling with feelings of stigma, prejudices and loss of control Giving up hope of recovery Accepting loss of control Living with fear	Experiencing people’s negative reaction to illness (Struggling with feelings of stigma and prejudices) Resignation, being powerless Being a burden to others Living with fear
5.	Hearing confirms existence and identity—experiences from persons with presbyacusis (Karlsson Espmark & Hansson Scherman, 2003)	**Identity** Conversation takes away or maintains identity It’s other people’s fault that I can’t hear Other people make you realize you can’t hear Society makes you think you shouldn’t mind about your hearing loss It’s natural to hear badly when you are old You should hear well all your life You want to keep a feeling of continuity in your daily life in spite of your hearing loss You don’t need to hear everything	Trying to hide one’s hearing impairment during conversations Blaming others for one’s hearing impairment Experiencing some people’s negative reactions to hearing impairments Experiencing society’s lack of interest in hearing problems among the elderly Everybody hears poorly when they get old Striving to live a normal life in spite of hearing impairment	Preserving identity and self-esteem as a normal person (Trying to hide illness) Difficulties in accepting being ill Experiencing people’s negative reactions to illness Feeling neglected by healthcare and society Limited participation in society
		**Existence** You want to hear so that you feel you’re alive You want to hear so you understand and keep yourself informed	Hearing is feeling alive Hearing is vital in order to be able to participate in society	
6.	Perceptions of health-related quality of life of men and women living with coeliac disease (Hallert et al., 2003)	**Bodily sensations** Physical endurance Bowel symptoms	Lacking physical endurance Constantly aware of stomach	Constantly being aware of own body Fatigue Restrictions in social participation Necessity of understanding own body
		**Social consequences** Value of food Identification Roles	Realizing that food can be social prerequisite Requiring special attention	Needing to take control Experiencing people’s negative reactions to illness Requiring special attention
		**Coping strategies** Acceptance Control	
7.	Patients’ experiences of physical limitations in daily life activities when suffering from chronic heart failure: a phenomenographic analysis (Pihl et al., 2011)	**Need of finding practical solutions in daily life** Demanding to change the character of activities Needing to continuously plan the activities of daily life Needing support to manage activities of daily life	Realizing the need to find practical solutions in daily life	Adapting to new life situations Fear Limited capacity to live everyday life Fatigue Disrupted identity (Losing one’s social role) Being a burden to others
		**Having realistic expectations about the future** Assuming a need for change in daily life Striving to maintain the quality of daily life Continuously making progress in daily life	Having realistic expectations about the future	
		**Not believing in one’s own ability** Failing to realize their own physical capacity and fear preventing them from performing the activities in daily life	Not believing in one’s own ability	
		**Losing one’s social role in daily life** Clear lack of important content in daily life Being unable to manage important activities in daily life	Feeling lack of content due to fatigue, resulting inability to perform important activities in daily life	
8.	Male patients with congestive heart failure and their conceptions of the life situation (Martensson et al., 1997)	**Feeling a belief in the future** **Gaining awareness** **Feeling support from the environment** **Feeling limitation** **Feeling lack of energy** **Feeling resignation**	Feeling a belief in the future Gaining awareness Reaching for support Experiencing limitations Feeling lack of energy Feeling resigned	Adapting to new life situations Resignation Fatigue Feeling limitations in everyday life Need for support network (family)
9.	Female patients with congestive heart failure: how they conceive their life situation (Martensson et al., 1998)	**Feeling content** Content with one’s past life Content with one’s present life situation	Feeling content with life	Adapting to new life situations Need for support network (family)
		**Feeling sense of support** Feeling of abandonment Sense of devotion	Feeling vulnerable Needing unconditional support	Feeling limitations in everyday life Fear and anxiety Being a burden
		**Feeling a sense of limitation** Physical restriction Social restriction	Feeling physically and socially restricted	Feeling worthless Resignation (feeling powerless)
		**Feeling anxiety** Insecurity in relation to one’s self Insecurity in relation to those in their surroundings	Becoming insecure	
		**Feeling powerless** Sense of worthlessness Sense of being a burden	Being worthless	
10.	Stress in women’s daily life before and after a myocardial infarction: a qualitative analysis (Sjostrom-Strand & Fridlund, 2007)	**Personal traits** Restlessness Prolonged anxiety Having a meaningless life Conditions that trigger ill health Being preoccupied with thoughts	Constantly feeling anxious Feeling stressed by necessary life style changes Feeling stressed by constant brooding	Fear and anxiety Adaptation to new life situation Lack of peace of mind Limited capacity to live everyday life Financial stresses
		**Effects of the immediate surroundings** Being depressed and lonely Long-term frustration Being stretched to the limit Demanding responsibilities Financial burden	Feeling stressed by not being able to carry out normal duties as before Feeling stressed by financial status	
11.	Womens’ experience of a myocardial infarction: 5 years later (Sjostrom-Strand et al., 2011)	**Consequences of a myocardial infarction** Fear and anxiety for the future Suffering from other serious illness Medication and secondary effects	Feeling fear and anxiety for the future Worrying about secondary effects of necessary medication	Fear and anxiety Lack of peace of mind Fatigue Feeling neglected by healthcare and society
		**Adjustment to a new life situation**: Rehabilitation Fatigue and other health complaints Fearing another MI Downgrading Moving forward with difficulties Gratefulness Taking responsibilities for lifestyle changes The recovery process Interaction with family and friends Being aware of the heart Financial stress	Worrying about lack of rehabilitation Experiencing overwhelming fatigue Fearing another MI Feeling neglected by health care professionals Feeling ambivalent about living as before Feeling thankful for being alive Taking responsibility for lifestyle changes Needing support of family and friends Being constantly aware of having a heart Feeling stressed by financial status	Need for support network (family) Constantly being aware of own body Financial stresses Adaptation to new life situation
12.	Adapting to living with a mechanical aortic heart valve: a phenomenographic study (Oterhals et al., 2013)	**The competent patient** Well-informed about factors that influence INR (International Normalized Ratio) levels Want to perform self-monitoring of INR Prevent infections Recognizes body’s signals	Being well-informed about INR levels Wanting to self-monitor INR Trying to prevent infections Recognizing body signals	Necessity of understanding own body Being aware of own body Needing to take control Adapting to new body and treatment situations Fear, anxiety and worry Feeling neglected by healthcare professionals
		**The adjusted patient** Accepted and adapted to the clicking sound of the valve Were back to their normal life Emphasizes the importance of exercise and rehabilitation Adjusted to the oral anticoagulation treatment Bruises and bleedings unproblematic	Accepting and adapting to sound of heart valve Returning to normal living Being aware of importance of exercise and rehabilitation Adjusting to oral anticoagulation treatment Not worrying about bruises and bleeding	
		**The unaware patient** Lacks knowledge about effects food and alcohol can have on INR levels Missing postoperative information Not aware of preventing endocarditis	The dissociated patient: Lacking knowledge about effects of food and alcohol on INR levels Missing postop information Not being aware of importance of preventing endocarditis	
		**The worried patient** Taking warfarin influences everyday life Annoyed by the clicking sound of the valve Worried about possible future consequences of the disease Fear of complications Fears inadequate follow-up by GP Hides surgery-related scars	Feeling influences of warfarin on daily living Feeling annoyed by clicking of heart valve Worrying about possible future consequences of illness Fearing complications Worrying about inadequate follow-up by GPs. Hiding surgery-related scars	

### Phase 4. Determining how the findings of the selected articles are related and translating them into one another

The translation proceeded by comparing the findings with each other, and noting that key findings from individual articles were present in more than one article.

### Phase 5. Synthesizing the translations to produce a new interpretation

The key ideas and phrases were translated into each other and synthesized to produce our phenomenographic interpretation, which identified four ways of experiencing chronic illness, with four distinct differences in focus and meaning.

## Findings

**Figure 1:**
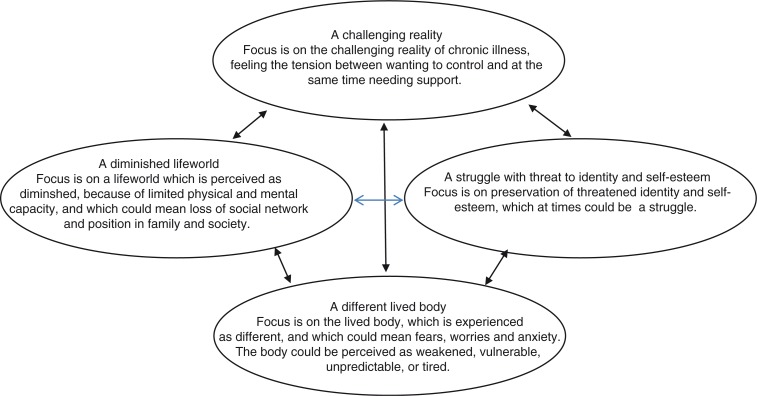
Outcome Space. The four categories of description of ways of experiencing chronic illness.

Four categories describing patients’ experiences of chronic illness were identified: a different lived body, a struggle with threat to identity and self-esteem, a diminished lifeworld, and a challenging reality. They are presented in the outcome space (Figure 1). These findings are the result of the development of new interpretations that go beyond the original interpretations in the individual articles included in the synthesis. They give a holistic perspective on the experience of chronic illness, in which the categories, seeming to be part of a process, continue to influence each other over time.

### A different lived body

In this category, focus was on the lived body, which was experienced as different, and which could mean fears, worries, and anxiety. The body could be experienced as weakened, vulnerable, unpredictable, or tired.

An illness which had affected the heart, one of the most vital organs in the body, could be experienced as living in a weakened body. These patients were preoccupied with what had happened to them, and the perceived weakness of their bodies. Living under these conditions lacked meaning and joy, and became instead a question of resting and sleeping, fear and worry, including even fear of death (Pihl, Fridlund, & Martensson, 2011; Sjostrom-Strand, Ivarsson, & Sjoberg, 2011). Patients with coronary heart disease, after one myocardial infarction, feared recurrence (Sjostrom-Strand & Fridlund, 2007; Sjostrom-Strand et al., 2011), whereas patients living with a mechanical heart valve feared infections and rejection of the valve (Oterhals, Fridlund, Nordrehaug, Haaverstad, & Norekval, 2013).

The body as weakened could be experienced by patients living with a leg ulcer as well, although perhaps not to the same extent as patients with heart conditions. However, the thoughts of these patients could be preoccupied with their own safety, and fears of injury and pain (Roaldsen, Biguet, & Elfving, 2011).

An asthma/allergy or coeliac disease could be experienced as living in a vulnerable body. Patients suffering from these conditions were very conscious of the vulnerability of their body to external factors. They could be reminded of their body when they come into contact with specific triggering factors in the external environment (Scherman, Dahlgren, & Lowhagen, 2002). For patients with coeliac disease, the body was vulnerable to certain food, and many women experienced as well persistent bowel symptoms such as diarrhoea and pain, even though they were very careful to adhere to a gluten-free diet (Hallert, Sandlund, & Broqvist, 2003).

For patients with epilepsy, the body could be experienced as unpredictable (Raty & Wilde-Larsson, 2011). These patients could feel the need to always be on guard for signals of an arising seizure. They lived as well with the fear of physical injury related to seizures, and also of the side-effects of the so vital seizure medications.

Many patients experienced their illness as living in a tired body. Patients with chronic renal failure experienced profound mental and physical fatigue (Heiwe, Clyne, & Dahlgren, 2003). These patients could have difficulties in performing everyday activities, from personal hygiene to normal household chores. The physical fatigue, so hard to overcome, could result in fear of falling when walking.

### A struggle with threat to identity and self-esteem

In this category, patients focused on their identity, which they perceived was threatened. For these patients, the reactions of other people toward their symptoms, as well as their own reactions to these symptoms, were experienced as a threat to their true and genuine identity (i.e., to be seen as the individuals they felt themselves to be), which they then struggled to preserve.

Some asthma patients tried to deny their illness, even to themselves, by interpreting their symptoms as normal reactions (Scherman et al., 2002). A few tried to hide their disease by keeping it secret, wanting to appear to be in good health. Others chose instead to “negotiate,” deciding to accept their attacks, and not lose out on the enjoyment of valued activities. This could mean choosing to prioritize the rules of the social and cultural world over the rules of the medical world. They fought against being identified as a sick person. These patients took part in activities which were practiced by everybody, such as keeping fit—in contrast to physiotherapy—so as not to be classified as ill. They also made a clear separation between body and mind, in the sense that they could look upon their body as an apparatus with a fault in its construction, a fault which they personally were not responsible for.

What motivated some patients with venous leg ulcer to perform physical training was primarily the wish to retain the identity they had had as healthy people, to remain normal (Roaldsen et al., 2011). Treatment strategies were looked upon as ways to distract pain. For instance, compression stockings could threaten their identity as a normal person, and therefore were avoided in the company of other people. In this manner, their hiding of symptoms resembled patients with asthma.

Patients with hearing impairment (presbyacusis) developed different strategies to maintain their genuine identity, such as restricting their conversation to situations where their identity was already established (Karlsson Espmark & Hansson Scherman, 2003). Just as patients with asthma and leg ulcer patients, they tried to hide their symptoms. They also denied their hearing problems by blaming their difficulties on the unclear pronunciation of others. The restrictions of society on hearing aids were experienced as a threat to their identity as a full member of society. Simultaneously, some patients could perceive their deteriorating hearing as a natural part of the ageing process, especially when meeting old friends who also had difficulties with hearing. In that context, their impairment could be included in their self-image and thereby strengthened their identity.

Patients with epilepsy who had a positive attitude toward their illness focused on trying to live a normal life (Raty & Wilde-Larsson, 2011), and felt no shame about their illness, i.e., did not try to hide it. This led to better control of the effects of seizures and more relaxed attitudes. Afraid of being perceived as “not normal,” patients with a negative attitude, on the other hand, appeared to focus on the condition. They felt dependent, questioned, and misjudged, and struggled with prejudices and stigma.

Females with coeliac disease seemed to identify themselves as coeliacs more often than males, but did not feel respected as such (Hallert et al., 2003). They expected others to take their dietetic needs in consideration, but experienced little understanding from their social network. The male coeliacs seemed to be less bothered. They appeared to distance themselves from identifying with a special illness, and preferred to include family members in their own diet.

### A diminished lifeworld

In this category, focus was on a lifeworld which was experienced as diminished, and which could mean loss of social network, position in family, and society.

Almost all patients with heart conditions experienced that their capacities in daily life had become limited (Martensson, Karlsson, & Fridlund, 1997, 1998; Pihl et al., 2011; Sjostrom-Strand & Fridlund, 2007). The perceived weakened body prevented these patients from daily physical and social activities, and limited their working capacity. They experienced a lack of important content in daily life, which included both physical and mental activities, because of their inability to participate in available activities.

Patients with coeliac disease experienced restrictions in their social life, not being able to participate in full because they constantly had to be on watch, controlling whatever food they were offered (Hallert et al., 2003). Aware that food and eating symbolize the spirit of community (togetherness), they felt “different” in the company of others. Women, especially, seemed to define themselves as “coeliacs,” feeling relaxed only when socializing with other people with the same disease. However, they longed to participate in their usual network, and to be shown consideration for their handicap.

Patients with hearing problems sometimes preferred to restrict their social life to family and close friends, as conversation with strangers demanded special strategies (Karlsson Espmark & Hansson Scherman, 2003). Patients with epilepsy sometimes thought that the best strategy was concealment and avoiding exposure, i.e., withdrawal (Raty & Wilde-Larsson, 2011). In addition, patients with epilepsy endured the constant control of society, as they had limited rights as members of society (certain insurances, driver's license, choice of profession).

### A challenging reality

Focus in this category was on the reality of living with chronic illness, which could be challenging for the patients, as it could mean feeling the tension between wanting to control, while at the same time needing support and help from others.

Some asthma/allergy patients made efforts to control their illness by distancing themselves from the medical perspective, i.e., becoming less compliant, and trying to find their own ways (Scherman et al., 2002). Some leg ulcer patients experimented with the amount of physical activity and pain killers to find out what could be beneficial for them (Roaldsen et al., 2011). Although they sometimes felt uncertain, they chose to trust themselves.

Two different patterns of living were discerned among the epilepsy patients (Raty & Wilde-Larsson, 2011). One pattern meant living a normal life, gaining and maintaining control. This included taking responsibility in relation to seizures, i.e., trying to prevent them, protecting self and others in case of a seizure. The other pattern meant living with focus on the illness, with restrictions in ways of living, and being ruled by the illness.

Patients with a hearing loss could control their social situation with the help of people who knew about their problem, and who helped them participate in conversations. Or, they prepared themselves in different ways, for instance when going to the theatre they could inform themselves about the story beforehand (Karlsson Espmark & Hansson Scherman, 2003). They sometimes downgraded previous interests by denying the importance of hearing in specific contexts, and also changed their interests to activities that did not require hearing. Retreating to others with hearing problems was a way to feel relaxed and understood.

Because eating gluten-free food was the only way to remain healthy, patients with coeliac disease were forced to adapt their food intake (Hallert et al., 2003). They were careful to control their food, and females especially did not trust other people's statements, but wanted see for themselves that the food was “safe.”

For patients with chronic heart failure a careful planning of daily activities was important for being able to live the life they wanted (Pihl et al., 2011). Patients living with a mechanical aortic heart valve, disturbed by the clicking sound of the valves, tried to minimize their observation of this sound and mask the sound of the valve, for instance, by listening to music (Oterhals et al., 2013). Other heart patients, living in a weakened state, could develop an increased dependence on healthcare providers. These patients feared being neglected by healthcare professionals. Women could have worries about not being taken seriously, and felt looked down upon by doctors who seemed to be uninterested and even arrogant (Sjostrom-Strand et al., 2011). Men could fear inadequate follow-ups. They were not sure their GPs (General Practitioners) had sufficient knowledge, and feared that complications would not be noticed (Oterhals et al., 2013; Sjostrom-Strand et al., 2011). These patients also experienced a strong need for support from family and friends (Martensson et al., 1997, 1998; Sjostrom-Strand et al., 2011). Support could be practical or psychological (Sjostrom-Strand et al., 2011). Some female patients found that they could no longer contribute to the support of their family (Sjostrom-Strand & Fridlund, 2007). They felt worthless and worried about becoming a burden instead. Such feelings could make it difficult to ask for or receive necessary help and support. Women who had had a myocardial infarct took responsibility for lifestyle changes (such as quitting smoking) but found it difficult to manage everything all by their own (Sjostrom-Strand et al., 2011). They wanted support and follow-up by healthcare.

Some leg ulcer patients relied heavily on support from staff. They depended on strict prescriptions on how to exercise, and on encouragement from the staff in order to engage in training.

## Discussion

Some common chronic diseases are not included in this meta-synthesis, such as diabetes and cancer, as our meta-ethnographic synthesis is limited to 12 articles on patient's experiences of chronic illness which had used a phenomenographic approach. However, the findings described above are consistent with many articles which describe the commonalities and variations in the ways that individuals experience chronic illness. As an example, profound fatigue can be felt by patients with cancer, diabetes, SLE (Systemic Lupus Erythematosus), MS (Multiple Sclerosis), chronic obstructive pulmonary disease, and chronic renal failure (Paterson, Thorne, & Russell, 2002; Stridsman, Lindberg, & Skar, 2014; Thorne & Paterson, 2000). Threats to identity and self-esteem have been reported by patients living with a spinal cord injury, HIV infection, and hearing loss (Paterson et al., 2002). The experience of a chronic illness can be disease specific. It can differ if an illness is life-threatening (i.e., heart disease) versus chronic but not terminal (i.e., coeliac disease), if it is characterized by periods of unpredictability (i.e., asthma) versus gradual progression (diabetes), or if it happens to be traditionally discredited, for example epilepsy, versus a disease that is considered common, such as heart disease or diabetes (Thorne & Paterson, 2000). In general, the experience of chronic illness, for the patient, involves first responding to the effects of the illness, followed by adaptation and management (Charmaz, 1995; Paterson, Thorne, & Dewis, 1998).

The main contribution of our meta-ethnographic synthesis and phenomenographic interpretation to knowledge of patients’ experiences of their chronic illnesses is primarily that the different ways of experiencing are related, and how they can influence each other. For instance, a positive or negative change in how patients experience that they can carry out daily activities can influence their experience of their lived body. This can generate consequences in how they experience the scope of their lifeworld, which in turn may lead to changes in how they experience their identity and self-esteem. A practical example of a positive loop is the following: A patient acquires a practical aid which facilitates her outdoor mobility. This leads to feelings of physical vitality, which affect her self-esteem and self-image. Thus, all these experiences of chronic illnesses, here described in four categories, are reciprocally interdependent. Any change can lead either to a positive or negative spiral. This is an extremely important perspective for healthcare staff to have in mind when planning different actions together with patients, and, for patients as well, when contemplating what is happening in their lives.

The four categories seem to be represented in all the disease groups except the renal failure and heart disease groups, which do not indicate any aspects of identity. However, it is difficult to believe that patients with heart disease do not reflect on how living with a life-threatening disease might influence their social roles, self-esteem, and identity. The lack of material regarding identity might then depend on incomplete analyses, or too narrow scopes of these specific studies, and indicate rather methodological issues than no perceptions at all of identity matters among the heart or kidney patients. Another explanation might be that identity issues were overshadowed by the struggle for pure survival. For heart patients, death anxiety was salient, as they were constantly aware of the risk of having a new heart attack. The renal failure patients felt an overwhelming fatigue that was described as being “hollow inside,” “only a kind of shell that's functioning” or a feeling of “actually not being there.” This can be interpreted as a feeling of being left with no identity. Thus, questions about identity might be secondary to body survival.

In her comprehensive work on people with chronic diseases, Charmaz (1995) discusses adapting as one mode of living with impairment and loss of bodily function. She defines adapting as “altering life and self to accommodate to physical losses and to reunify body and self accordingly.” People would “adapt as they regain a sense of wholeness, of unity of body and self in the face of loss.” In a similar work Aujoulat, Marcolongo, Bonadiman, and Deccache (2008) concluded that most individuals with chronic diseases were struggling with two dimensions of identity work: 1) the process of separating their identity as an ill person from previous or “preferred identity” (holding on), and 2) integrating their identity as an ill person into a coherent whole by “ letting go” and thereby learning to identify and accept both the limits and possibilities linked to their disease (Aujoulat et al., 2008). This is to reach a reconciled self.

In our meta-synthesis, it is possible to find traces of this kind of adapting among some of the participants in the selected articles. However, this aspect was not directly observed by the authors of these articles. Although the initial experiences of a different body could be perceived as a threat to a patient's identity, the struggle to preserve identity seemed to continue to dominate throughout the process, as patients’ primary motives in “wanting to control” still focused on the need to protect their genuine identity, self-esteem, and integrity, to be seen as the individuals they felt themselves to be. Our findings suggest that this process is not final but seems to reiterate itself in recurring loops, depending on various life events and the progress of the disease.

### Critical reflections

Issues and questions that arose during the process of our synthesis need to be noted. The first issue involved the use of phenomenography in healthcare research to date. In reviewing the articles, we found that the way the results were presented varied a great deal. Only two articles presented an outcome space which described the structural relation between the categories (Heiwe et al., 2003; Roaldsen et al., 2011). Three other articles discussed a relationship between categories, and by that suggested an outcome space in prose (Raty & Wilde-Larsson, 2011; Scherman et al., 2002; Sjostrom-Strand et al., 2011). The remaining authors instead presented their findings in tables (Hallert et al., 2003; Karlsson Espmark & Hansson Scherman, 2003; Oterhals et al., 2013; Pihl et al., 2011; Sjostrom-Strand & Fridlund, 2007), and others just presented some categories (Martensson et al., 1997, 1998). No effort was made to suggest the order and logical relation between the different categories. This reduces the very point of phenomenography, and might as well be the results of a content analysis. This suggests the need for a better understanding and application of phenomenography if this method is to be useful in healthcare research.

What about the quality of our own study? The ways of experiencing chronic illness are presented as categories in an outcome space. These categories have not formed a hierarchy, nor have they been defined in terms of increasing complexity. Instead, they have been interpreted as part of a process with recurring loops, where the different ways of experiencing continue to influence each other over time. According to Marton and Booth (1997), 1) “the individual categories of ways of experiencing should each stand in clear relation to the phenomenon so that each category tells us something distinct about a particular way of experiencing the phenomenon,” 2) the categories “have to stand in a logical relationship with one another,” and 3) “the system should be parsimonious” (p. 125). We argue that our categories of description and their relationships fulfill these criteria and express qualitatively distinct ways of experiencing chronic illness. The categories may appear to be more detailed than expected in traditional phenomenographic studies. This is partly because of our ambition to report in detail the material that this synthesis is based on. They also mirror the phenomenon in study, the experience of chronic illness, which is heterogeneous and multifaceted.

Another issue that arose involved the suitability of phenomenography in qualitative healthcare research. Marton has continuously emphasized that phenomenography is aimed at questions of relevance to learning and understanding in an educational setting, and that it is not psychology (Marton, 1996). With psychology, he seems to mean cognitive psychology. His views on issues like emotions, motives, and social psychology including norms and human relationship are not evident. However, he has described three lines of phenomenographic research: one that concerns content-related studies of more general aspects of learning, another that focuses on studies of learning within particular content domains, for instance mathematics, and finally one line which is focused on “the description of how people conceive of various aspects of their reality.” He stresses that this “corresponds more to a ‘pure’ phenomenographic knowledge interest” (Marton, 1986). Dall'alba (2000), in referring to this, points out that most phenomenographic research thus far has been done within the first two lines.

Giorgi (1999) doubted that the approach should be exclusively tied to pedagogics and suggested that it would be possible to widen the field of application of phenomenography with some adaptations. Such an extension would make necessary a more precise clarification of the approach. We agree. In its original form, it is evident that a hierarchical relation between descriptive categories suits a pedagogic aim well, both with regard to learning, competence, and professional development (Sandberg, 2000). However, in our present study we found that a phenomenographic approach gave a psychologically trustworthy and vivid picture of how chronic illness can be experienced by people with such diseases. In such contexts—describing emotional, motivational, relational, affective, and so forth ways of experiencing a phenomenon—a hierarchical relation between categories does not seem appropriate. This was also exemplified by the articles included in our meta-synthesis; none of them came up with a hierarchy. In our present synthesis, the relation between categories was interpreted as a reciprocal process which could reiterate itself in recurring loops. Lessening the demand for a hierarchical relationship might allow a fruitful expansion of the method. Thus, we think that the phenomenographic approach of exploring different ways of experiencing a phenomenon is valuable also in clinical contexts, and that other relations between ways of experiencing, besides hierarchies, can be useful in understanding various phenomena within healthcare and probably other settings.

## Conclusions

This meta-ethnographic synthesis with a phenomenographic interpretation of the material, based on interviews with 148 patients presented in 12 selected articles, found that patients’ experiences of chronic illness can be described in terms of a different lived body, a struggle with threat to identity and self-esteem, a diminished lifeworld, and a challenging reality. These experiences relate to each other in a process with recurring loops, where the different ways of experiencing continue to influence each other over time. Our synthesis has thus provided a holistic perspective on patients’ experiences of their illness; in this way, new knowledge has been added to the field.

According to our findings, phenomenography has the potential to add to the understanding of patients’ experiences of chronic illness. Patients may benefit from seeing that their illness can be experienced in many different ways and that it has many aspects, which then can lead to a more comprehensive understanding of their illness. With a better understanding of patients’ reactions, professionals can contribute to and enhance patients’ empowerment process. Against this background, we find it is worthwhile to expand the scope of the phenomenographic method outside the field of pedagogics. This presupposes a revision of the application to include a wider and more comprehensive description of the different ways illness and healthcare phenomena can be experienced, and how these different ways are related to each other, with less focus on hierarchical relations.
